# Metabolic correlates to critical speed in murine models of sickle cell disease

**DOI:** 10.3389/fphys.2023.1151268

**Published:** 2023-03-13

**Authors:** Francesca I. Cendali, Travis Nemkov, Christina Lisk, Ian S. Lacroix, Seyed-Mehdi Nouraie, Yingze Zhang, Victor R. Gordeuk, Paul W. Buehler, David Irwin, Angelo D’Alessandro

**Affiliations:** ^1^ Department of Biochemistry and Molecular Genetics, University of Colorado Denver, Aurora, CO, United States; ^2^ Department of Pulmonology, University of Colorado Denver, Aurora, CO, United States; ^3^ Division of Pulmonary, Allergy and Critical Care Medicine, Department of Medicine, University of Pittsburgh, Pittsburgh, PA, United States; ^4^ Department of Medicine, University of Illinois at Chicago, Chicago, IL, United States; ^5^ Department of Pathology, University of Maryland, Baltimore, MD, United States; ^6^ Center for Blood Oxygen Transport, Department of Pediatrics, Baltimore, MD, United States

**Keywords:** critical speed, sickle cell disease, exercise, metabolism, carnitines

## Abstract

**Introduction:** Exercise intolerance is a common clinical manifestation in patients with sickle cell disease (SCD), though the mechanisms are incompletely understood.

**Methods:** Here we leverage a murine mouse model of sickle cell disease, the Berkeley mouse, to characterize response to exercise *via* determination of critical speed (CS), a functional measurement of mouse running speed upon exerting to exhaustion.

**Results:** Upon observing a wide distribution in critical speed phenotypes, we systematically determined metabolic aberrations in plasma and organs—including heart, kidney, liver, lung, and spleen—from mice ranked based on critical speed performances (top vs. bottom 25%). Results indicated clear signatures of systemic and organ-specific alterations in carboxylic acids, sphingosine 1-phosphate and acylcarnitine metabolism. Metabolites in these pathways showed significant correlations with critical speed across all matrices. Findings from murine models were thus further validated in 433 sickle cell disease patients (SS genotype). Metabolomics analyses of plasma from 281 subjects in this cohort (with HbA < 10% to decrease confounding effects of recent transfusion events) were used to identify metabolic correlates to sub-maximal exercise test performances, as measure by 6 min walking test in this clinical cohort. Results confirmed strong correlation between test performances and dysregulated levels of circulating carboxylic acids (especially succinate) and sphingosine 1-phosphate.

**Discussion:** We identified novel circulating metabolic markers of exercise intolerance in mouse models of sickle cell disease and sickle cell patients.

## Highlights

• Murine models of sickle cell disease display a wide range of critical speed measurements after exercise challenge.
• Top vs. bottom 25% mice based on critical speed show different metabolic phenotypes in plasma and multiple organs.
• Alterations in the circulating and tissue levels of carboxylic acids and acyl-carnitines correlate with critical speed measurements in these mice and 6 min walk test in humans.

## 1 Introduction

In the United States, approximately one in every 500 African American and one in 36,000 Hispanic American newborns suffer from sickle cells disease (SCD) (MacMullen and Dulski, 2011), a condition caused by a single nucleotide substitution in the gene coding for the hemoglobin beta chain (HBB). This mutation results in the expression of a sickle Hb (HbS) allele β^S^, the mutant protein being referred to as sickle β-globin owing to an amino acid substitution (D6V in HbS). HBB is an essential component of the hemoglobin tetramer, the most abundant protein in the mature erythrocyte (250–270 million copies/cell (Bryk and Wiśniewski, 2017)), which is essential to the red cell critical role in oxygen transport and off-load through the body (Nemkov et al., 2018). Under hypoxic conditions, such as those observed in peripheral capillaries when oxygen demand is high [e.g., in response to exercise (Xu and Rhodes, 1999; Radak et al., 2013)], deoxygenated HbS can crystallize into hemoglobin macrofibers (McDade and Josephs, 1993) and cause sickling of the erythrocyte, with red cell morphology that switches from the canonical discocytic shape to assume a crescent or sickled shape. Carrying either one or two copies of the mutated gene results in sickle cell trait (2 million people in the United States) or SCD (100,000 people in the United States) (MacMullen and Dulski, 2011), respectively. Between 300,000 and 400,000 neonates are diagnosed with SCD globally each year (Kato et al., 2018). Carrying two copies of HbS is associated with more severe clinical manifestations, such as hemolytic anemia, inflammation and recurrent vaso-occlusive episodes (Rees et al., 2010). In children, SCD manifests itself with the development of cerebrovascular disease and cognitive impairment, which requires lifelong reliance on transfusion therapies (Rees et al., 2010). Age-associated comorbidities are exacerbated in the SCD patient, who usually suffers from recurrent episodes of vaso-occlusion and inflammation as a result of progressive damage to most organs, including the brain, kidneys, lungs, bones, and cardiovascular system (Rees et al., 2010).

Alterations of systems metabolism (especially plasma) in SCD have long been associated with increased susceptibility to oxidant stress (Darghouth et al., 2011; Zhang et al., 2014; Gehrke et al., 2019; Demblé et al., 2020b; Ilboudo et al., 2021). These studies suggested that depletion of glutamine, a precursor to glutamate for the biosynthesis of the main reducing equivalent in mature RBCs, glutathione—is a potential contributors to the increased susceptibility to hemolysis of the sickle erythrocyte (Ilboudo et al., 2021). While glutamine supplementation studies in this population are currently underway (Niihara et al., 2018), it is unclear whether the approach would be sufficient to cope with oxidant stress in the sickle cell patient (or even just subjects with sickle cell traits), especially in response to stimuli that further exacerbate it, such as exercise (Radak et al., 2013; Pietrangelo et al., 2015). Indeed, oxidant stress is elevated in sickle RBCs, which ultimately manifests itself with damage to proteins and lipids (Wu et al., 2016; Nader et al., 2020). The Lands cycle (Wu et al., 2016) is the main pathway through which the mature erythrocyte leverages circulating carnitines to repair oxidatively damaged lipids to preserve RBC membrane deformability, owing to the incapacity to synthesize *de novo* long chain fatty acids and complex lipids (Himbert et al., 2021). Unmitigated oxidant stress to the erythrocyte ultimately triggers intravascular (Kato et al., 2017) and extravascular hemolysis through splenic sequestration and erythrophagocytosis by the reticuloendothelial system (Brousse et al., 2014). When chronic, this phenomenon promotes increases in splenic macrophage levels of iron, as well as in circulating levels of iron and heme (Buehler et al., 2021a; Buehler et al., 2021b), all factors mediating cardiovascular (Kato, 2012) and renal effects in the sickle cell population (Gladwin et al., 2014). Low exercise capacity is observed in patients suffering from SCD, as gleaned by a 6 min walk test, a simple cardiopulmonary functional testing modality that allows for a non-specific, integrated assessment of the many systems involved during physical activity (Matos Casano and Anjum, 2022). However, limited efficacy observed upon interventions aimed at mitigating endothelial dysfunction through purinergic agonists such as sildenafil (Machado et al., 2011), or by stimulating the expression of alternative hemoglobin isoforms (e.g., fetal hemoglobin) *via* hydroxyurea treatment (Wali and Moheeb, 2011). As such, identification of molecular markers of poor exercise tolerance in SCD can inform on novel interventional strategies in this patient population.

In response to exercise, increased O_2_ demand is balanced by compensatory regulation of cardiovascular function and O_2_ transport capacity by mechanisms of metabolic regulation [.g., 2,3-diphosphoglycerate and adenosine triphosphate in the red cell (Donovan et al., 2021), or sphingosine 1-phosphate (Sun et al., 2016) or adenosine (Liu et al., 2016) in plasma], which promote the stabilization of deoxyhemoglobin and oxygen release from the erythrocyte (Rifkind et al., 1991). While these mechanisms represent beneficial adaptations to boost performance under physiological conditions (Rabcuka et al., 2022), in the pathological sickle erythrocyte, HbS stabilization of under (exercise-induced) hypoxic conditions and oxidant stress can trigger sickling and facilitate cell lysis (Wu et al., 2016). Indeed, even subjects with sickle cell traits, who carry a single copy of HbS, are characterized by metabolic alterations linked to impaired fatty acid and mitochondrial metabolism (Nemkov et al., 2022), and are cautioned against strenuous exercise, especially under hypoxic conditions (e.g., high altitude) (Tripette et al., 2009; Ferguson et al., 2019).

In the present study, we investigated functional implications to exercise performance by leveraging a murine model of critical speed (CS), linking increased tissue oxygen demand in response to exercise to dysfunctional systems metabolism in plasma and multiple organs. Critical speed (CS) is a functional measurement of mouse running speed upon exerting to exhaustion (Billat et al., 2005). Here, Berkeley mice, a well-established transgenic murine model of SCD whereby human HbS is expressed (Manci et al., 2006)—completed a series of treadmill-based exercise tolerance tests (Buehler et al., 2021b). These tests were performed on a custom-built motor-driven treadmill following a 5 days acclimatization period in which animals were familiarized with treadmill running for 5 min/day at speed of 5 m/min up a 5% incline. For the last several runs, speed of the treadmill will be increased progressively over the last minute to ∼10–20 m/min to familiarize the mice with high-speed running. Endurance capacity and CS, i.e., the asymptote of the speed to duration relationship (Billat et al., 2005; Buehler et al., 2021b), was determined in each animal after a total of 5 runs to exhaustion on a treadmill (performed with a minimum 48 h recovery separation period). By combining metabolomics data with CS measurements in Berk mice, we provide a preliminary overview of the metabolic derangements underpinning normal or sub-normal exercise performance in the context of SCD. We thus compare our results to human data on 6 min walk test in the WALK PHASST cohort, identifying carboxylic acid, fatty acid and acyl-carnitine metabolism as critical correlates to physical exertion in the context of SCD.

## 2 Materials and methods

### 2.1 Plasma metabolomics in the WALK-PHASST cohort

Subject enrolling, eligibility and exclusion criteria were extensively described in prior publications (Machado et al., 2011; Sachdev et al., 2011; Milton et al., 2013; Nouraie et al., 2013; Gladwin et al., 2014; Saraf et al., 2014). A total of 587 plasma samples were obtained from SCD patients for metabolomics analyses. Clinical covariates were collected prior to metabolomics analysis, including 6 min walk test (Kato, 2012; Johnson et al., 2021). Samples were there selected on the basis of the genotype (SS) and percentage of hemoglobin S (sickle—HbS) in high performance liquid chromatographic assay; additionally, samples from patients with hemoglobin A > 10% were excluded, to account for any confounding effects from donor blood, leaving 433 samples for the present metabolomics analysis. Description of these measurements had been previously described (Machado et al., 2011; Sachdev et al., 2011; Milton et al., 2013; Nouraie et al., 2013; Gladwin et al., 2014; Saraf et al., 2014). Samples were then stored at −80°C until further processing. All experimental protocols were approved by the University of Pittsburgh, as part of the WALK-PHaSST clinical trial (NCT00492531 at ClinicalTrials.gov and HLB01371616a BioLINCC study).

### 2.2 Animals

Eight- to ten-week-old female Berk-SS mice were either obtained from Jackson Laboratories (Bar Harbor, ME, United States) or our in-house Berk SCD mouse colony. Mice were housed and bred in an AAALAC accredited animal facility at the University of Colorado, Denver, Anschutz Medical campus and were maintained on a 12:12 light-dark cycle with food and water available *ad libitum*. Female heterozygous Berk-SS mice were bred with male homozygous Berk-SS mice to generate homozygous offspring. Specifically, Berk-SS mice with genotype Tg(Hu-miniLCR α1 Gγ Aγ δ βs) Hba0/0 Hbb0/0 and the hemizygous with genotype Tg(Hu-miniLCR α1 Gγ Aγ δ βs) Hba0/0 Hbb0 Hbb+ were littermates (Pászty et al., 1997). Genotyping of mice used for breeding and experiments was performed by TransnetYX (Cordova, TN, United States). A total of 12 mice Berk-SS mice (both males and females) were used in the present investigation and after determination of exercise tolerance were sorted into the top 25% (*n* = 3) and bottom 25% (*n* = 3). Levels of discomfort and distress were monitored daily by the in-house animal care staff, with a veterinarian available as needed. Mice presented no pain or discomfort associated with hypoxia and were alert as well as eating, drinking, and grooming normally while housed. All experimental procedures were conducted under the guidelines recommended by The Journal of Physiology (Buehler et al., 2021a), the National Institutes of Health and were approved by the Institutional Animal Care and Use Committee at the University of Colorado, Denver, Anschutz Medical Campus (protocol no. 00218—“Vascular and organ effects of Cell free hemoglobin”). Mice were humanely euthanized by exsanguination and cervical dislocation.

### 2.3 Treadmill exercise and constant speed tests

Critical speed (CS) studies were performed as previously described (Buehler et al., 2021b). Prior to the determination of CS, mice completed a treadmill familiarization phase, which consisted of four ∼5 min runs on a motor-driven rodent treadmill (Exer 3/6, Columbus Instruments, Columbus, Ohio, United States). For the first several runs, the treadmill speed was maintained at 10–15 m/min (up a 5° grade, which was maintained throughout all treadmill tests). For the last several runs, the speed of the treadmill increased progressively over the last minute to ∼30–35 m/min to familiarize the mice with high speed running. Animals were encouraged to run with intermittent bursts of compressed room air aimed at the hind limbs from directly above the animal (so as not to push the mouse up the treadmill). All treadmill testing protocols were designed and conducted by experienced staff and strictly followed the guidelines set by the American Physiological Society’s resource book for the design of animal exercise protocols (Jones, 2007).

The CS was determined using a modified version of the methodology used by Copp et al. (2010) for rats (Copp et al., 2010), as well as following the guidelines set forth by Poole et al. (2020). After completion of the treadmill familiarization period, each mouse performed 3–5 runs to exhaustion, in random order, at a constant speed that resulted in fatigue between 1 and 15 min (speeds ranging from 30 to 50 m/min). Each test was performed on separate days with a minimum of 24 h between tests. For each constant-speed trial, mice were given a 2-min warm-up period where they ran at 15–20 m/min followed by a 1-min period of quiet resting. To start the test, the treadmill speed was increased rapidly over a 10 s period to the desired speed at which point a stopwatch was started. Testing was terminated and time to exhaustion was measured to the nearest tenth of a second whenever the mouse could no longer maintain pace with the treadmill despite obvious exertion of effort. A successful constant-speed test was determined if 1) the mouse could quickly adapt to the treadmill speed at the beginning of the test (e.g., did not waste energy), 2) a noticeable change in gait occurred preceding exhaustion (i.e., lowering of the hindlimbs and rising of the snout), and 3) the animal’s righting reflex was markedly attenuated when placed on their back in a supine position (an unexhausted quadruped will typically attempt to right themselves within ∼1 s).

### 2.4 Data modeling for the determination of CS

Following successful completion of the constant speed treadmill tests, the CS and finite distance capacity (D′) were calculated for each mouse using the linear 1/time model (Speed = D' × 1/time + CS) as described previously (Ferguson et al., 2019; Ferguson et al., 2020; Buehler et al., 2021a). In this model, the treadmill speed used for the constant speed test is plotted as a function of the inverse of time to exhaustion and the *y*-intercept of the regression line yields the CS and the slope is the D'.

### 2.5 Metabolomics

Metabolomics analyses were performed as previously described (Nemkov et al., 2019). Briefly, a volume of 10 μl of frozen plasma or 20 mg of ground tissue (GenoGrinder) was extracted in either 90 μl or 1 ml, respectively of methanol:acetonitrile:water (5:3:2, v/v/v). After vortexing at 4°C for 30 min, extracts were separated from the protein pellet by centrifugation for 10 min at 10,000 g at 4°C. Ultra-High-Pressure Liquid Chromatography-Mass Spectrometry analyses were performed using a Vanquish UHPLC coupled online to a Q Exactive mass spectrometer (Thermo Fisher, Bremen, Germany).

## 3 Results

### 3.1 Critical speed varies significantly among mice with sickle cell disease

**FIGURE 1:**
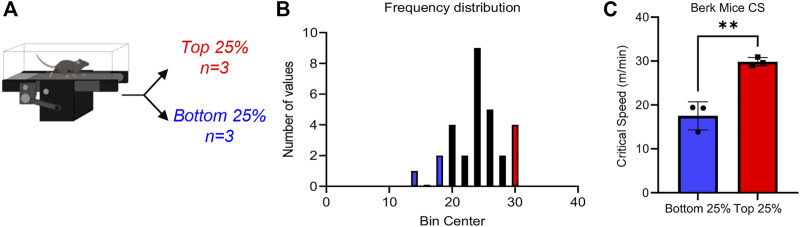
Critical speed overview Sickle Cell mice underwent critical speed test, then were separated into Top 25% (*n* = 3) and Bottom 25% [*n* = 3—**(A)**]. In **(B)**, frequency distribution of mouse critical speed. Blue represents Bottom 25% and red represents Top 25% (*n* = 3) selected for the study. In **(C)**, top 25% critical speeds compared to bottom 25% critical speed. Unpaired *t*-test yields a significance of .0031.

CS measurements were performed in 12 Berkeley mice (Pászty et al., 1997), i.e., transgenic mice expressing human α^−^, γ^−^, and β^S-^globin (Figure 1A). Results indicate a wide spread range of CS measurements across all mice tested in this study, with median CS of 24.0 m/min (Figure 1B), lower than values reported in healthy control mice (∼30 m/min (Buehler et al., 2021a)) with the same genetic background (i.e., STOCK background of the triple mutant strain is a mixture of FVB/N, 129, DBA/2, Black Swiss and >50% C57BL/6 genomes, further backcrossed to C57BL/6J for additional generations). However, a wide spread of CS measurements was observed in this population (standard deviation of 4.2 m/min, i.e., ±17.5%), suggesting heterogeneous responses to physical exercise challenge in this murine model of SCD (Figure 1B). Specifically, when focusing on the top and bottom 25% mice by CS, we observed two significantly different groups (Figure 1C), with the low CS group in the range of 17 m/min and the high CS group in the 30.9 m/min (i.e., comparable to normal values).

### 3.2 Metabolic characterization of plasma and organs in Berkeley mice with low or normal critical speed

**FIGURE 2:**
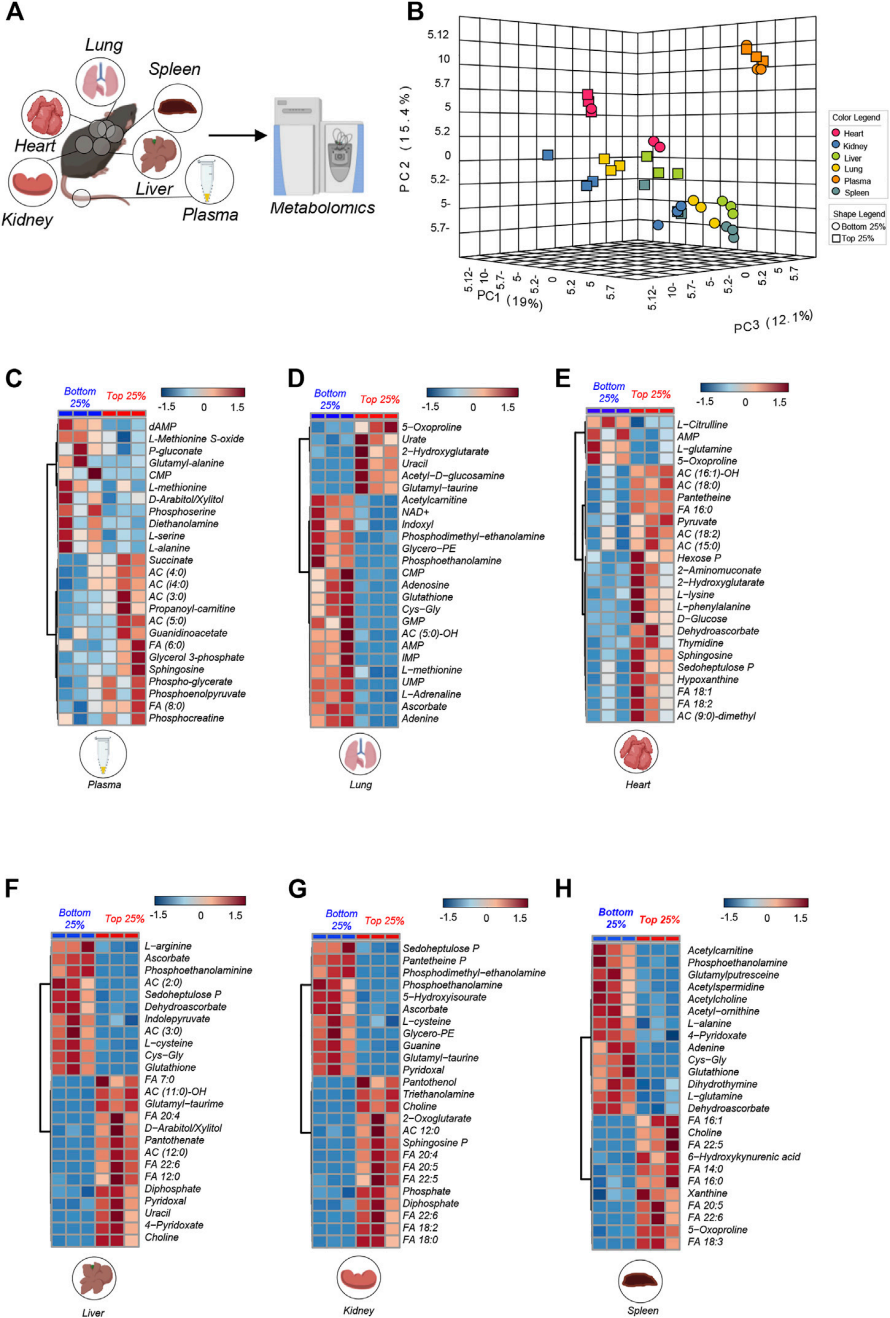
Metabolic overview highlights difference in matrixes. In **(A)** All matrix (plasma, spleen, liver, lung, heart, kidney) run on mass spectrometry for metabolite analysis from the top 25% (*n* = 3) and bottom 25% (*n* = 3) mice. In **(B)** PCA analysis of all matrixes analyzed in Top 25% (*n* = 3) and Bottom 25% (*n* = 3). In **(C–H)**: Hierarchical clustering of the top 25 ANOVA significant metabolites across multiple organs, as specified at the bottom of each heat map.

Increased oxygen and metabolic demand sustain exertion during physical activity. To investigate the metabolic underpinning of low or high (sub- or normal critical speed) in Berkeley mice, we selected the top 3 and bottom 3 Berkeley mice based on CS measurements, and performed metabolomics analyses of plasma and multiple organs, including heart, kidney, liver, lung and spleen (Figure 2A). Principal component analysis (PCA—Figure 2B) shows that, on top of the expected matrix specific-effects on the metabolome, breakdown by CS was evident especially in the organs (heart, kidney, lung, liver, spleen). Indeed, low vs. normal CS mice were separated across PC1, which explained 19% of the total variance across all samples (Figure 2B).

To provide an overview of the top metabolites that varied in each tissue on the basis of CS, we performed hierarchical clustering analyses and generated heat maps of the top 25 metabolites by fold change and *t*-test between the two groups (low vs. normal CS—for plasma, lung, heart, liver, kidney, spleen in Figures 2C–H, respectively). Results are also shown in the form of volcano plots (high in normal CS in red and high in low CS group in blue—Supplementary Figure S1). These analyses revealed a significant accumulation of free fatty acids (FA) in most tissues from normal CS mice, especially liver, heart, kidney and spleen (Figure 2), suggestive of ongoing lipid oxidation. Similarly, some matrices (plasma and heart) showed elevation of acyl-carnitines (AC) in the normal CS group (Figure 2). Berk mice with low CS were characterized by higher plasma and organ levels (e.g., liver and lungs) of metabolites involved in redox responses, such as glutathione and sulfur homeostasis (including glutathione, 5-oxoproline and other metabolites of the gamma-glutamyl cycle, methionine and methionine sulfoxide, cysteine, ascorbate, and pentose phosphate pathway metabolites, 6-phosphogluconate and sedoheptulose phosphate). Higher levels of amino acids (alanine, arginine, glutamine) were elevated in mice with low CS. On the other hand, we observed elevated sphingosine 1-phosphate, succinate, glycolytic metabolites (hexose phosphate, phosphoglycerate, phosphoenolpyruvate) in organs and plasma from normal CS mice.

### 3.4 Metabolic correlates to critical speed in plasma and multiple organs

**FIGURE 3:**
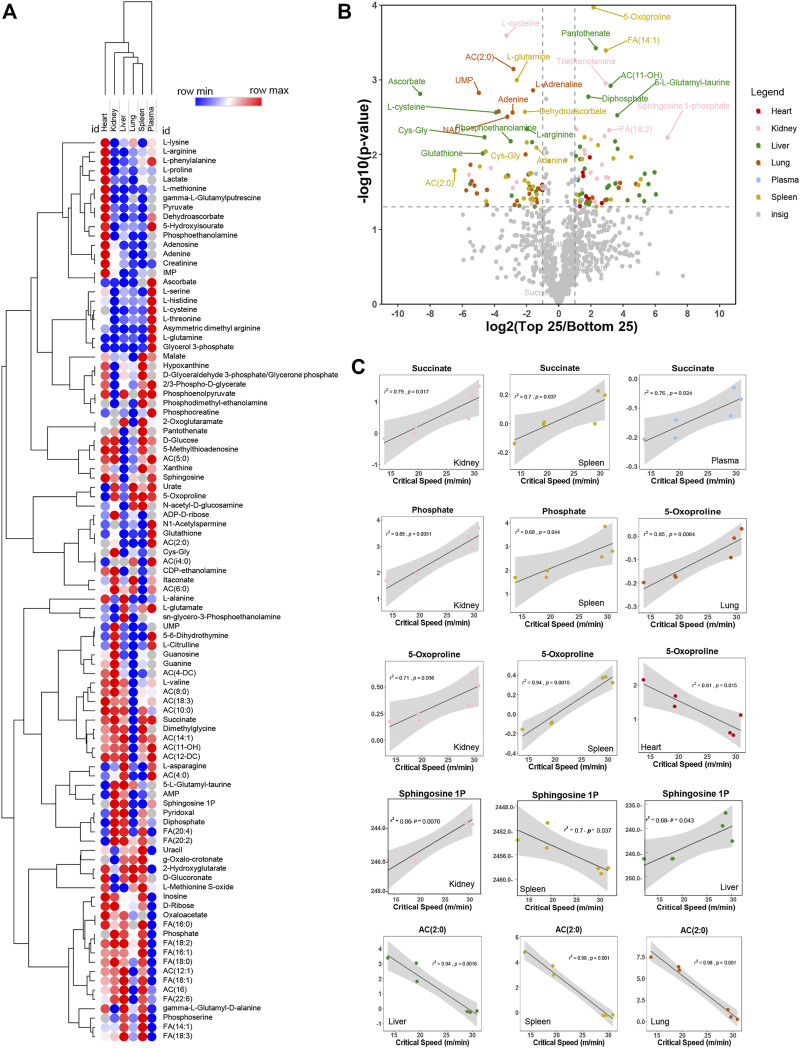
Metabolites correlate negatively and positively with critical speed. In **(A)**, heat map of top correlations to bottom correlations with critical speed. In **(B)**, volcano plot exhibiting metabolite levels that were increased in Top25 or Bottom 25 CS mice. The *x*-axis shows log2 transformed average fold changes of Top25/Bottom25 CS mice. While the *y*-axis shows -log10 transformed *p*-values for each corresponding fold change. Dashed vertical lines are positioned at ± x = 1 [log2(2) = 1, so fold change = 2] and the horizontal line is positioned at y = 1.3 [-log10(0.05)]. Metabolites showing log2 transformed fold changes greater than 1, and -log10 transformed *p*-values greater than 1.3 were considered significantly enriched in Top25 CS mice. Metabolites showing log2 transformed fold changes less than −1, and -log10 transformed *p*-values greater than 1.3 were considered significantly enriched in Bottom25 CS mice. Significantly enriched metabolites were color coded based on the originating matrix type, as indicated in the legend. In **(C)**, linear regressions for significant hits as identified in the volcano plot both for negative correlation and positive correlation.

We thus performed correlation analyses of metabolomics data in plasma and multiple organs. Results are reported in the form of a heat map of correlations for the most significant metabolic correlates to CS across all organs (Figure 3A). Metabolites were color coded for each organ and correlations were shown in the form of a volcano plot in Figure 3B, with the *x*-axis representing log2 fold changes in the ratios of top vs. bottom 25% CS mice and *y*-axis indicating the -log10 of *p*-value of this ratio. A similar analysis was repeated in Supplementary Figure S2, where the *x*-axis indicated the Spearman correlation between CS and metabolites, color coded again following the same scheme and summarized in tabulated form in Supplementary Figures S2A, B, respectively). Pathway analysis of the top hits from this elaboration showed a significant enrichment in metabolites involved in transamination reactions (alanine, aspartate, glutamate), glutathione metabolism and tricarboxylic acid (TCA) cycle—Supplementary Figure S2C. Top correlates for each organs are reported in the form of scatter plots (with interpolated linear correlation curves for each panel), including succinate (in kidney, spleen and plasma), phosphate (kidney and spleen), 5-oxoproline (positively correlated in lung, kidney and spleen; but negatively correlated in heart), sphingosine 1-phosphate (kidney, spleen and liver) and acetyl-carnitine (AC 2:0—negative correlate in liver, spleen and kidney—Figure 3C). Altogether, these analyses reveal that metabolic differences in the kidney liver, spleen were mostly positively correlated with CS, while changes in the lung and heart were negatively correlated with CS.

### 3.5 Metabolic correlates to 6 min walk test in plasma of patients with sickle cell disease

**FIGURE 4:**
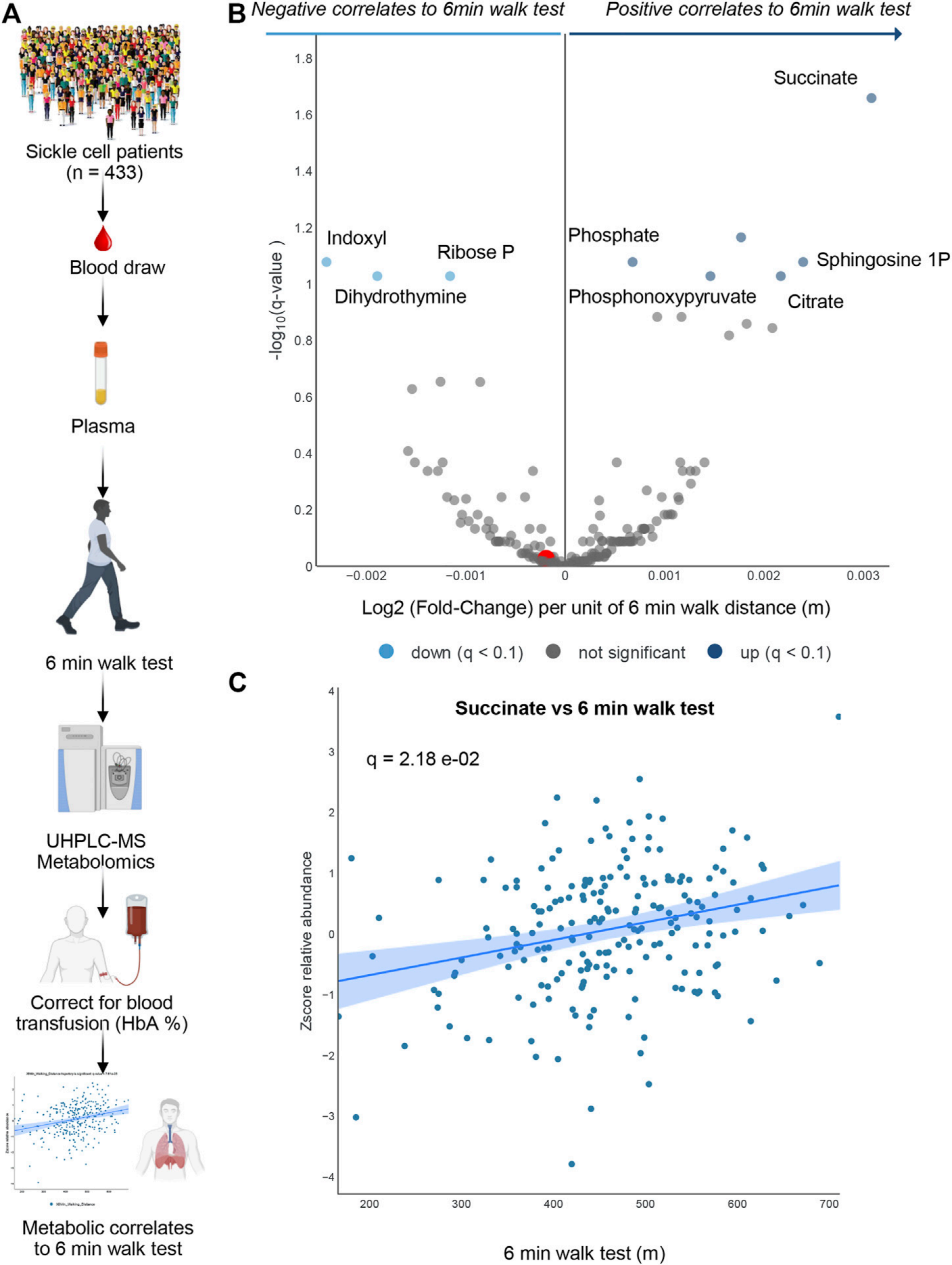
Human Sickle Cell patients 6-min walk test. In **(A)** Overview of the experimental design for 6 min walk test in a cohort of sickle cell patients. In **(B)** volcano plot for metabolites correlating 6 min walk test. In **(C)** A positive Succinate correlation plot with 6-min walk test.

To determine whether the metabolomics findings in mice could be recapitulated, at least in part, in humans with SCD, we performed metabolomics analyses in plasma from 433 patients with SCD (SS genotype). In this original cohort, 281 patients had HbA < 10% (Figure 4A). These patients are part of the WALK PHASST SCD cohort, previously characterized at the clinical level for parameters including 6 min walk test (Gladwin et al., 2014), a simple cardiopulmonary functional test to probe systems responses to physical activity (Demir and Küçükoğlu, 2015; Matos Casano and Anjum, 2022). A volcano plot of correlation analyses (*x*-axis represents the log2 fold change per unit of 6 min walking distance in meters, while *y*-axis represents the -log10 of *p*-value—Figure 4B) shows a strong positive association between multiple carboxylic acids, including succinate (a scatter plot of plasma succinate levels vs. 6 min walk test is shown in Figure 4C) and citrate, as well as sphingosine 1-phosphate, consistently with observations in mice (Figure 4B). Of note, top negative correlates (Figure 4B) included the tryptophan metabolite of bacterial origin, indoxyl, as well as ribose phosphate (the final product of the pentose phosphate pathway—consistent with observations of higher levels of intermediates of this pathway in the low CS mice) and dihydrothymine (higher in low CS mice as well).

## 4 Discussion

Exercise intolerance is a common comorbidity in patients with SCD, with a spectrum of disease severity phenotypes in part explained by decreased pulmonary capacity (VO_2_ max < 80%), altered cardiac reserve (Hammoudi et al., 2022) and anemia (van Beers et al., 2014). Multiple studies have pinpointed at the metabolic underpinnings of an increased susceptibility of red blood cell (RBC) hemolysis in the context of SCD (Darghouth et al., 2011; Zhang et al., 2014; Bakshi and Morris, 2016; Wu et al., 2016; Kato et al., 2017; Culp-Hill et al., 2018; Adebiyi et al., 2019; Dembélé et al., 2020a; Morris et al., 2020; Buehler et al., 2021a) with intravascular hemolysis representing a now well-established etiological contributors to cardiopulmonary dysfunction in the pathophysiology of SCD (Kato et al., 2017). However, the link between systemic metabolic derangements and exercise intolerance in this patient population remains unexplored. Interventional studies aimed at mitigating the cardiovascular clinical manifestations in patients with SCD (e.g., *via* promotion of vasodilation through sildenafil) have adopted 6 min walk test to determine improvements in exercise intolerance (Machado et al., 2005; Rao et al., 2011). Here we show for the first time that positive correlation exists between the plasma metabolome and 6 min walk test in a large cohort of SCD patients. Specifically, we show a significant positive correlation between the circulating levels of carboxylic acids (especially succinate) and negative correlation with circulating levels of pentose phosphate metabolites (ribose phosphate) with 6 min walk test in patients with SS genotype who have not recently received blood transfusion (i.e., HbA < 10%). Circulating levels of succinate increase in response to pathological hypoxia [e.g., following ischemia (Chouchani et al., 2014) or hemorrhage (Dʼalessandro et al., 2017)] or exercise in humans (San-Millan et al., 2020; Nemkov et al., 2021) and animal models (Cavalli et al., 2017). This metabolite is an indicator of mitochondrial dysfunction following a blockade of complex II and can fuel the formation of reactive oxygen species through reverse electron transfer upon reoxygenation/reperfusion (Chouchani et al., 2014; Pell et al., 2016; Prag et al., 2020). Acidification of organellar pH in response to exercise activity results in protonation of succinate to a monocarboxylate form, which makes it a substrate for monocarboxylate transporters to the cytosol and to the extracellular compartment, increasing circulating levels of this otherwise intracellularly compartmentalized dicarboxylate (Reddy et al., 2020). Of note, succinate levels have been shown to exert immunomodulatory functions (Ryan et al., 2019), by promoting inflammatory events such as the synthesis of interleukin-1 upon the stabilization of its upstream transcriptional regulatory, hypoxia-inducible factor 1 alpha (Tannahill et al., 2013). Circulating succinate can also promote neutrophil sequestration in the lung and acute respiratory distress syndrome in critically ill patients (Nunns et al., 2020). Therefore, elevation in circulating levels of succinate in SCD patients following a bout of exercise may further worsen the clinical manifestation of inflammatory comorbidities in this population.

Elevated levels of sphingosine 1-phosphate were observed in SCD patients with the longest 6 min walk distance. This is interesting because this metabolite is elevated following erythrocyte-specific synthesis *via* the enzyme sphingosine kinase 1 in response to (high altitude) hypoxia (Sun et al., 2016). Increases in red cell sphingosine 1-phosphate are deleterious in the context of SCD, in that this metabolite cooperatively contributes to the 2,3-dipshophoglycerate-dependent stabilization of deoxyhemoglobin (Sun et al., 2017). While this adaptation is beneficial in the context of physiological [altitude (Sun et al., 2016)] or pathological hypoxia [e.g., COVID-19 (Thomas et al., 2020)], stabilization of the deoxygenated form of HbS favors sickling.

We also report a significant negative correlation between 6 min walk test and the levels of indoxyl, a tryptophan metabolite of bacterial origin and—in its sulfate form—an uremic toxin that is elevated in patients with SCD (Konopelski and Ufnal, 2018; Dutta et al., 2019). Though preliminary, this result may be suggestive of a role of systemic inflammatory complications and gut dysbiosis in the pathology of exercise intolerance in the SCD population. Notably, ribose phosphate—the end product of the pentose phosphate pathway—was significantly negatively correlated with 6 min walk test in patients with SCD. This is interesting because this metabolite is mostly cytosolic, which suggests that elevated circulating levels may be attributable to red cell hemolysis. Indeed, elevated production of ribose phosphate *via* the pentose phosphate pathway is a mechanism that erythrocytes leverage to counteract oxidant stress *via* generation of NADPH, a critical reducing equivalent involved in glutathione homeostasis and redox cycling of many antioxidant systems in the mature erythrocyte [reviewed in (D'Alessandro et al., 2019)]. Genetic mutations leading to hypomorphic activity of the rate limiting enzyme of the pentose phosphate pathway, G6PD deficiency is the most common enzymopathy in humans, as it affects ∼500 million people around the world (Luzzatto et al., 2020). As G6PD deficiency blunts red cell antioxidant responses (D'Alessandro et al., 2021) and primes them for increased intra- (Page et al., 2021) and extra-vascular hemolysis (Francis et al., 2020), it has been speculated that G6PD deficiency—which is not uncommon in sickle cell populations as it may have co-evolved to protect from malaria (Luzzatto et al., 2020)—may play a role in the etiopathology of cardiovascular manifestations of SCD that are at least in part explained by an impairment of red cell antioxidant defense systems (Karafin et al., 2018; Esperti et al., 2022).

Regulation of RBC antioxidant capacity by G6PD, the rate-limiting enzyme of the pentose phosphate pathway, is in part co-operating with a mechanism of oxygen-dependent metabolic modulation (Lewis et al., 2009), whereby deoxyhemoglobin binding to the N-terminus cytosolic domain of band 3—the most abundant membrane protein in RBCs—outcompetes glycolytic enzymes that are bound to and inhibited at this very same residue (Campanella et al., 2005; Issaian et al., 2021). At high oxygen saturation, binding of glycolytic enzymes to band 3 reduces fluxes through glycolysis, promoting the activation of the pentose phosphate pathway by low of mass action (D'Alessandro et al., 2012; Rinalducci et al., 2015; Reisz et al., 2016). However, this mechanism of oxygen dependent metabolic modulation is impaired in sickle RBCs (Rotter et al., 2010; Rogers et al., 2013; Rogers et al., 2021), suggesting an incompletely understood interplay between G6PD status, band 3-hemoglobin (A and S) interaction and antioxidant status of the RBC (Chu et al., 2016; Martin et al., 2022), which warrants further investigations.

Animal models of SCD like the Berkeley mouse (Pászty et al., 1997) have long been shown to recapitulate hemolytic disease (Manci et al., 2006), cardiovascular dysfunction and exercise intolerance (Buehler et al., 2021a; Buehler et al., 2021b) in a tractable murine system. Here we aimed at determining whether similar metabolic correlates to exercise intolerance are observed in this murine model than what observed in humans for the 6 min walk test, groundwork that will pave the way for interventional studies at the pharmacological and dietary level to mitigate this phenotype. The use of an animal model—especially one that is characterized, like patients with SCD, by a spectrum of critical speed measurements in the sub-physiological to physiological range compared to WT mice (Buehler et al., 2021a)—allowed us to investigate not just the circulating plasma metabolome, but also organ-specific derangements following a bout of exercise to exhaustion, an exploratory analysis that would be precluded in humans, for ethical and technical reasons. Overall, our data show a strong positive correlation between critical speed and circulating or organ specific-levels of carboxylic acids, especially succinate—confirming and expanding on observations in humans, which were merely based on plasma metabolome assays. Also confirming data in patients with SCD described above, slower mice were characterized by higher levels of redox metabolites involved in glutathione homeostasis, pentose phosphate pathway and methionine homeostasis. Most notably, plasma and organs from faster running mice were associated with higher levels of free fatty acids, suggestive of ongoing lipid oxidation. These data are consistent with previous studies on exercise metabolism, showing elevated fatty acid oxidation as the main driver of exercise performance especially in response to endurance physical activity (San-Millan et al., 2020; Nemkov et al., 2021), such as the case for the critical speed test (Billat et al., 2005). Of note, this very pathway is blunted in patients suffering from exercise intolerance upon recovering from COVID-19, a recently described condition now referred to as post-acute sequelae to COVID-19 (Guntur et al., 2022). A very interesting finding was that variance in critical speed measurements was associated with significant variability in organ metabolomes, with spleen, liver and kidney showing the strongest phenotypes with many metabolites showing positive correlation to critical speed, while heart and lung showed negative correlates amongst the most significant hits. Interestingly, increased susceptibility to red cell intra- or extra-vascular hemolysis (in the spleen or liver)—such as in the context of SCD—has been recently linked to alterations in the circulating levels of sphingosine 1-phosphate, free fatty acids and acyl-carnitines, ultimately driving kidney dysfunction (Xie et al., 2020; Bissinger et al., 2021; Xu et al., 2022). To this end, it is perhaps worth noting that opposite trends in relation to critical speed were observed for sphingosine 1-phosphate in liver and spleen, or 5-oxoproline—a byproduct of the gamma-glutamyl-cycle in response to protein amino acyl glutathionylation - in the heart vs. all the other matrices, suggestive that adaptations to hypoxia and redox homeostasis in SCD vary across tissue and that plasma or red cell-centric investigations of metabolism in this disease may provide an incomplete picture.

Our study holds several limitations. Metaboomics analyses were limited to Berkeley mice, with no analyses of WT counterparts. This choice was motivated by the decision to focus on metabolic dysregulation in SCD patients for which 6 min walk test data were available. While merely observational in nature, our study provides the first characterization of multi-organ metabolic correlates to critical speed, paving the way for pharmacological or dietary interventions to mitigate exercise intolerance in the context of this disease (e.g., glutamine supplementation to sustain glutathione synthesis and fuel mitochondrial metabolism; pharmacological interventions targeting sphingosine 1 phosphate synthesis and/or export). Future studies will expand on the present analyses, also including additional measurements of cardiovascular function beyond critical speed. Interesting signatures—such as the negative correlation between pentose phosphate pathway and exercise tolerance in SCD patients (for which unfortunately hematocrit levels were not accessible for meta-analysis) and mice—are suggestive of a potential, as of yet unexplored role of G6PD deficiency (Demir et al., 2009) in the context of exercise-induced oxidant stress (Radak et al., 2013; Cavalli et al., 2017), which will be the focus of follow up investigations.

## Data Availability

The original contributions presented in the study are included in the article/Supplementary Material, further inquiries can be directed to the corresponding authors.

## Supplementary material

SUPPLEMENTARY FIGURE S1 Significant metabolites for each Matrix. In **(A-F)**: Volcano plot of each matrix as indicated at the top of each plot, and highlighted key significant metabolites.

SUPPLEMENTARY FIGURE S2 Line plots. In **(A)**: Metabolite wide correlation with measured CS values was performed using the Pearson method. In **(B)**: Top (positive and negative) significant metabolite correlates with CS were highlighted by matrix type; colors correspond to legend in **(A)**. In **(C)**: A pathway enrichment map prepared from intraindividual normalized values from each matrix in both Top 25% and Bottom 25%.
