# Supplementary figures and images for: Metabolic correlates to critical speed in murine models of sickle cell disease
Source: Front Physiol. 2023 Mar 13;14:1151268. doi: 10.3389/fphys.2023.1151268 (PMC10053510; doi:10.3389/fphys.2023.1151268)

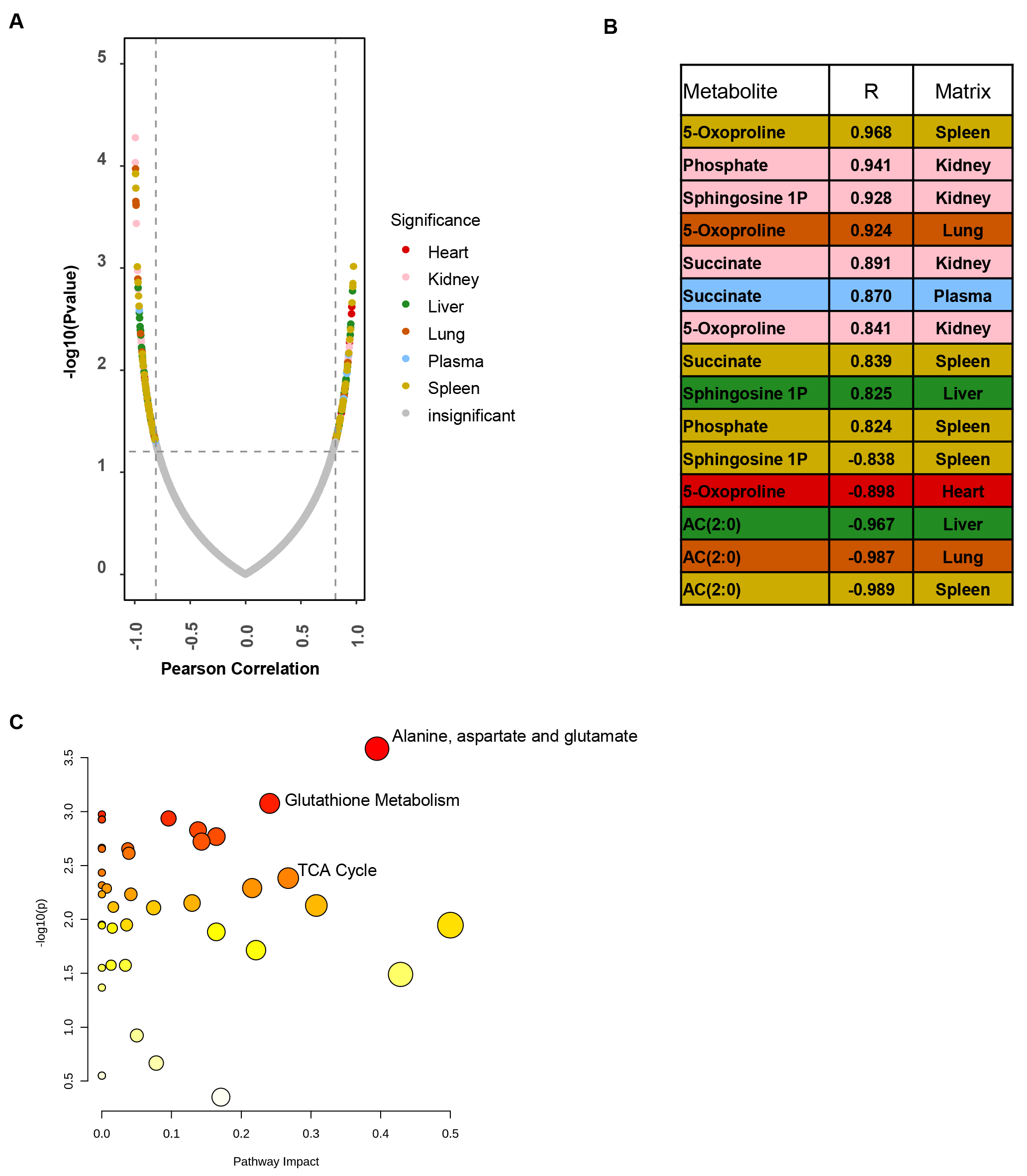

Supplement: Supplementary file 1 [file Image2.TIF]

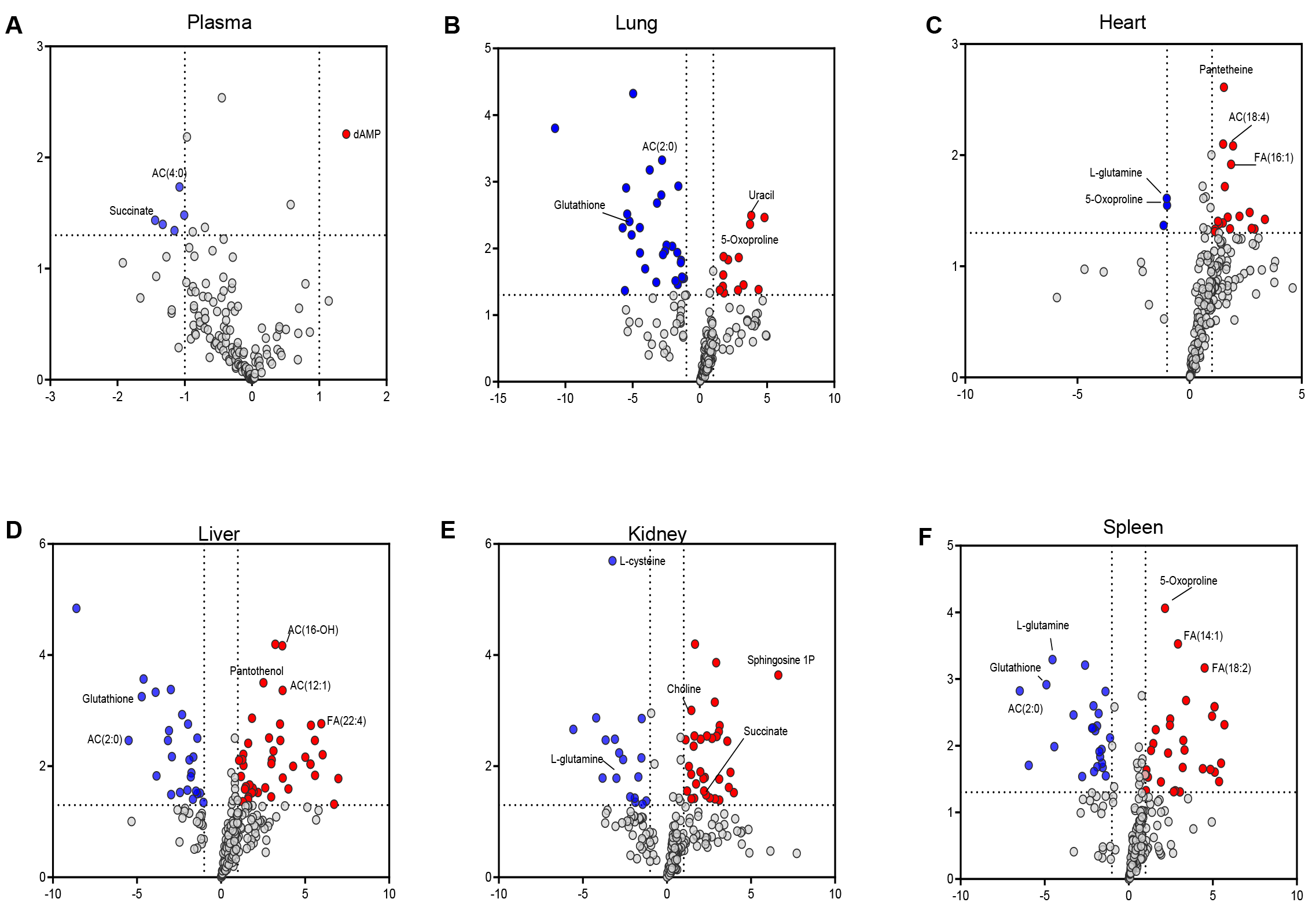

Supplement: Supplementary file 2 [file Image1.TIF]
